# Supplementary material for: Integrated β-catenin, BMP, PTEN, and Notch signalling patterns the nephron
Source: eLife. 2015 Feb 3;4:e04000. doi: 10.7554/eLife.04000 (PMC4337611; doi:10.7554/eLife.04000)
Supplement: Supplementary file 1. — Primers and UPL probes are used in qRT-PCR analysis. DOI: http://dx.doi.org/10.7554/eLife.04000.037 [file elife04000s001.pdf]

Supplementary Table1

Primers and UPL probes used in qRT-PCR analysis

**Genes**	**Primer (left)**	**Primer (right)**	**UPL Probe**
Tcf1	ggagctgcagccatatgatag	tagcctccttctctgccttg	96
Axin2	gagagtgagcggcagagc	cggctgactcgttctcct	96
Ccnd1	gagattgtgccatccatgc	ctcctcttcgcacttctgct	67
c-Myc	cctagtgctgcatgaggaga	tccacagacaccacatcaattt	77
Nphs2	ccatctggttctgcataaagg	ccaggacctttggctcttc	38
Synpo	gtagccaggtgagccaagg	ttttcggtgaagcttgtgc	67
Nphs1	aacatccagctcgtcagcat	agggctcacgctcacaac	45
Podxl	cctgcatctcactcccataat	tctgttgatgttgtggcactt	21
Fat1	caaccacgtcgaggaagc	caccgttgtctgtcacagtaactt	18
Fyn	gccaagcagtgtttgaaagg	cagcggtcggactgactt	89
Wnt4	ctggactccctccctgtctt	atgcccttgtcactgcaaa	62
Pax8	gcagctatgcctcttctgcta	gctgtaggcattgccagaat	4
Pax2	cactgatcctgcccacatta	cattagggacagagccctca	10
Fgf8	tcctgcctaaagtcacacagc	tgagctgatccgtcacca	32
Jag1	gaggcgtcctctgaaaaaca	acccaagccactgttaagaca	6
Lhx1	aatgcaacctgaccgagaag	cgcatttggtaccgaaacat	7
Dll1	gggcttctctggcttcaac	taagagttgccgaggtccac	103
HeyL	ctgaattgcgacgattggt	gcaagacctcagctttctcc	25
Hey1	accatcgaggtggaaaagg	cttctcgatgatgcctctcc	72
Lgr5 probe/primer1	cttcactcggtgcagtgct	gatcagccagctaccaaatagg	60
Lgr5 probe/primer2	ttcactcggtgcagtgct	ggatcagccagctaccaaata	100
Lgr5 probe/primer3	tgcagaacaaccagctgaga	gatgtggttggcatctaggc	69
Lgr4 probe/primer1	cagttaccagaagatgcatttaagaa	caaggcttttgggtggataa	20
Lgr4 probe/primer1	tcaggctattaaagcccttcc	tccatccgggataacagaaa	33
